# The risk of dietary multiple micronutrient inadequacies is widespread and geographically varied in Malawi

**DOI:** 10.1186/s40795-026-01369-2

**Published:** 2026-05-25

**Authors:** Gareth Osman, Elaine L. Ferguson, Lucia Segovia de la Revilla, Liberty Mlambo, Louise E. Ander, Edward J.M. Joy, Tinna Ng’ong’ola-Manani, Alexander A. Kalimbira

**Affiliations:** 1 Department of Human Nutrition and Health, Bunda College, Lilongwe University of Agriculture and Natural Resources, Lilongwe, Malawi; 2 Faculty of Epidemiology and Population Health, London School of Hygiene & Tropical Medicine, Keppel Street, London, WC1E 7HT UK; 3 School of Biosciences, University of Nottingham, Sutton Bonington Campus, Loughborough, LE12 5RD UK; 4 Department of Food Science and Technology, Bunda College, Lilongwe University of Agriculture and Natural Resources, Lilongwe, Malawi

**Keywords:** Inadequacy, Household Consumption and Expenditure Survey, Malawi, Micronutrients, Geographic variation

## Abstract

**Background:**

In resource-constrained settings, routine assessment of micronutrient deficiencies is often limited to a small set of priority nutrients due to the high cost and complexity of biomarker-based surveys. The objective of this study was to estimate the risk of inadequate apparent intake for 15 micronutrients in Malawi, disaggregated by wealth group and geographic location, using Household Consumption and Expenditure Survey (HCES) data.

**Methods:**

We used food consumption data from Malawi’s Fifth Integrated Household Survey (2019/20), matched with relevant food composition tables, to estimate apparent daily intake per adult female equivalent (AFE) – the reference individual used to apply micronutrient requirement cut-offs for 15 micronutrients: vitamins A, C, E, B1, B2, B3, B6, B9, B12, and minerals Ca, Cu, Fe, Mg, Se, and Zn. Intake estimates were compared against Harmonized Average Requirement values using a fixed cut-point method for all micronutrients except Fe, for which the full probability approach was applied.

**Results:**

Nationally, inadequacy was widespread, with 12 of the 15 assessed micronutrients showing prevalence above 20%. These included vitamins A, B2, B3, B6, B9, B12, C, and E, and minerals Ca, Fe, Se, and Zn. Vitamin B2 and Ca had the highest inadequacy rates, both exceeding 87%. Inadequacies were most pronounced in central region districts and among poorer, rural households. Diets dominated by cereals and limited food diversity contributed to these patterns. Geographic disparities were evident, with some districts facing substantially higher risks than others.

**Conclusions:**

The risk of dietary micronutrient inadequacies is widespread and geographically varied in Malawi. Expanding routine public health surveillance to include a wider range of micronutrients is essential for guiding targeted interventions and improving public health outcomes.

**Supplementary Information:**

The online version contains supplementary material available at 10.1186/s40795-026-01369-2.

## Background

Micronutrients – (vitamins and minerals) including iron (Fe), selenium (Se), zinc (Zn), and vitamins A, C, and the B-vitamins are vital for maintaining human health and physiological function [1]. Although required in small quantities, chronic inadequate dietary intakes of nutrient dense foods can lead to multiple, often concurrent deficiencies of these nutrients, with serious health and economic consequences, including impaired cognitive development, weakened immune function, maternal and child morbidity, and reduced labor productivity [2–4]. In many low- and middle-income countries, including Malawi, micronutrient deficiencies are primarily driven by suboptimal diets and low nutrient bioavailability [3, 5, 6]. In Malawi, for example, diets are typically dominated by cereal staples, such as maize, often complemented by starchy roots like cassava and sweet potatoes [7]. While these foods are energy-dense, their content of several bioavailable micronutrients is low, resulting in limited intake of critical vitamins and minerals.

Despite the importance of identifying populations at risk of low micronutrient intakes, Malawi has not conducted a national food consumption survey. Historically, three National Micronutrient Surveys (2001, 2009, and 2015/16) assessed a limited set of priority micronutrients using biomarker-based methods [8–10]. The 2015/16 National Micronutrient Survey (MNS) assessed six micronutrients – vitamin A, Fe, iodine, Zn, vitamin B12, and folate (vitamin B9), through biomarker analysis [10]. The survey was conducted during the El Niño–related 2015–2016 drought and peak food‑insecurity period, which reduced national food availability and likely affected dietary patterns and micronutrient risk at the time of data collection [11, 12]. Furthermore, the predominance of monotonous, cereal-based diets consumed in Malawi suggests that other micronutrient deficiencies beyond those routinely assessed might be common. Micronutrient biomarker surveys are expensive and logistically demanding to conduct, making it important to determine whether biomarker data on other critical micronutrients, potentially overlooked due to financial constraints are needed. Analyzing dietary data to estimate the population prevalence at risk of inadequate intakes across a wide range of micronutrients can inform such decisions.

Malawian diets are generally cereal-based, however, region or district-level differences in geography, climate, and cultural practices can influence access to and consumption of diverse food sources. For instance, proximity to Lake Malawi may increase access to fish and aquatic foods in lakeshore districts, while climatic conditions can influence the distribution of local crop production [13, 14]. In northern Malawi, cassava flour is a common staple, contrasting with the maize-dominated diets of the central and southern regions [15]. These variations in food availability and dietary habits suggest that the risk of inadequate intakes of micronutrients may not be uniform across the country, underscoring the need to assess whether district-level geographical differences in the risk of inadequate micronutrient intakes exist in Malawi.

In the absence of biomarker data, dietary assessment offers a practical alternative for identifying populations at risk of inadequate micronutrient intakes. While dietary data cannot confirm clinical deficiencies, it can highlight potential inadequacies that warrant further investigation and inform nutrition policy and programming. However, while individual-level dietary surveys, such as 24-hour recalls or weighed food records, are recommended for assessing dietary intakes and the prevalence of a population at risk of inadequate intakes, they are resource-intensive, time-consuming, and logistically demanding to implement at scale [16, 17]. This limits the collection of such data, in many countries, for comprehensive, population-wide dietary assessments. An alternative data source for dietary assessment is the Household Consumption and Expenditure Survey (HCES), referred to in Malawi as the Integrated Household Survey (IHS). These surveys are nationally representative and multicomponent, designed to capture a wide range of socioeconomic indicators, including household food consumption (i.e. using a predefined food list), for the purposes of poverty and welfare monitoring in a country. They are conducted by the National Statistical Office (NSO) in collaboration with the World Bank. By 2025, six rounds of the IHS had been completed in Malawi, typically on a five-year cycle. When paired with relevant food composition data, HCES can be used to estimate apparent nutrient intakes in each household – through comparison against a reference individual (e.g. adult woman) – to identify the percentage of households at risk of inadequate apparent intakes. Recent applications of HCES data in Malawi have informed population-level nutrient risk assessments and evaluated the potential impact of Large-Scale Food Fortification (LSFF) interventions, including vitamin A fortification of sugar and cooking oil, and the fortification of wheat flour with a standard premix containing vitamins A, B1, B2, B3, B6, B9, B12, Fe, and Zn [18–21].

In this study, we assessed a broader set of micronutrients than those typically included in national biomarker surveys and individual-level dietary assessments, using HCES data to estimate apparent intakes for 15 micronutrients: vitamins A, C, E, B1, B2, B3, B6, B9, B12, Calcium (Ca), copper (Cu), Fe, magnesium (Mg), Se, and Zn. Using HCES data, we estimated the risk of inadequate apparent intake of these micronutrients and explored their geographic variation at the district level. This study aimed to identify priority nutrients for future biomarker assessments and to support evidence-based nutrition programming and policy development in Malawi.

## Methods

### Food consumption data

We used household-level food consumption data from Malawi’s Fifth Integrated Household Survey (IHS5), which was a cross‑sectional survey conducted nationwide between April 2019 and April 2020 [22]. The survey employed a stratified two-stage sampling design, based on cartographic data (i.e. up-to-date geographic maps and enumeration area boundaries) and population figures from the 2018 Malawi Census. The final sample included 11,434 households, statistically representative at national, district, urban, and rural levels. Food consumption data were collected through the household questionnaire, specifically module_g1 (hh_mod_g1), which asked: *“Over the past 7 days*,* did you or others in your household consume any [food item]? How much in total did your household consume in the past 7 days?”* This 7‑day semi‑quantitative consumption module is not intended to estimate individual dietary intake, but it provides a reliable basis for deriving household‑level apparent nutrient availability, consistent with established HCES methodologies. Each household completed the 7‑day food consumption module once during its scheduled interview visit. The respondent was the adult household member most knowledgeable about food consumption; where necessary, other members were consulted to improve completeness. Responses were recorded for a predefined list of 135 food items. The interviews were administered by trained field enumerators from the NSO as part of the IHS’s standard multi‑topic data collection. Data collection was distributed proportionately across all areas throughout the year, helping to minimize seasonal bias in geographic comparisons.

To estimate daily apparent household food consumption, we divided the total quantity of each reported food item by seven. This daily quantity was then allocated proportionally across all household members using the Adult Female Equivalent (AFE) metric, in which each member is assigned an AFE value based on age‑, sex‑, and physiological‑status‑specific energy requirements. The sum of AFEs represents the household’s total energy‑demand units. Apparent intake for the reference individual (a non‑pregnant, non‑lactating adult female aged 18–29 years) was calculated by dividing the daily household food (or nutrient) quantity by the total household AFEs, consistent with previous studies [18, 19]. Apparent intake therefore reflects nutrient intakes approximated from household‑level food consumption data rather than directly measured individual consumption. The qualifier ‘apparent’ was used to distinguish these estimates from individual level dietary recall methods, and to serve as population‑level at-risk indicators.

### Calculation of Adult Female Equivalent (AFE) metric

We calculated apparent household food consumption and micronutrient intakes per day per AFE, following established methods as reported by Kalimbira et al. [18]. The AFE assumes that household food is distributed in proportion to the energy requirements of each household member, standardized to a nonpregnant, nonlactating, 18- to 29-year-old woman who served as a reference household member, i.e. 1 AFE [18, 19]. We calculated the AFEs by estimating age, sex, body weight and women’s physiological status (i.e. pregnant or lactating) for each household member based on FAO/WHO/UNU Human Energy Requirements guidelines [23]. For individuals under 18 years, we applied age- and sex-specific body weights and assumed a moderately active lifestyle [23]. For adults 18 years and above, we used average body weights derived from nationally representative data: 55 kg for women aged 18–29.9 years based on the 2015–16 Malawi Demographic Health Survey (MDHS) [24], and weights from a large-scale non-communicable disease survey for other age groups [25]. Specifically, we applied 65 kg for women aged 30–59.9 years and 60 kg for those aged ≥ 60 years. For men, average weights of 60 kg, 65 kg, and 60 kg were used for the 18–29.9, 30–59.9, and ≥ 60 years age groups, respectively. A moderate physical activity level (PAL) was assumed for all adults, corresponding to 1.75×BMR (basal metabolic rate). This PAL was selected to approximate the typical energy expenditure patterns observed in the general Malawian population [26].

Because the IHS5 dataset did not include direct information on lactation or breastfeeding status, we used age-specific breastfeeding prevalence rates from the 2015–16 MDHS to inform assumptions. Nearly all children under 12 months were assumed to be breastfed at some point, while prevalence rates of 90.5% and 76.6% were applied for children aged 12–17 months and 18–23 months, respectively. Breastfeeding status was randomly assigned based on these rates. For mothers of breastfed children, we added 330 kcal/day if the child was under 6 months and 400 kcal/day if the child was between 6 and 24 months [27]. These adjustments were only applied when breastfeeding was assumed. For breastfed children, we deducted estimated energy intake from breastmilk to determine their complementary food energy needs [28]. Pregnant women were assigned an additional 300 kcal/day [29]. The total number of AFEs per household was calculated by dividing the sum of individual energy requirements by the energy requirement of the reference woman. A worked example illustrating AFE allocation and proportional distribution of household food is provided in Additional Tables 1a and 1b.

### Food consumption data processing and micronutrient composition matching

We processed household-level food consumption data following established procedures adapted from Tang et al. and Adam et al. [19, 30]. All food quantities reported in both standard (e.g. milliliters) and non-standard units (e.g. pail, basin, heap) were converted to metric unit grams using region-specific conversion factors provided in the caloric conversion factor file in the IHS5 dataset. Where relevant, quantities were adjusted to reflect only the edible portions of foods by excluding inedible components. This was applicable to foods such as banana peels, skins of fruits and tuber, and bones of large fish that would have been discarded. We identified and managed potentially implausible values based on apparent food consumption per day per AFE by normalizing the quantities with the logarithmic transformation. Any food quantity exceeding five standard deviations above the mean of the log-transformed distribution was considered an outlier and replaced with the median quantity consumed among consumers of that food item [19].

We matched food items to available and relevant food composition data for energy and micronutrient profiling. The primary source was the 2019 Malawian Food Composition Table (FCT) [31], which accounted for most nutrient values (71%). Supplementary data were drawn from the Kenyan FCT − 16% [32], the Western African FCT – 12% [33], and the USDA Food Data Central database – 1% [34] for items not covered locally.

In line with Malawi’s national fortification policy, we assumed that all sugar and cooking oil were fortified with vitamin A, and wheat flour was fortified with a standard mix of micronutrients including vitamins A, B1, B2, B3, B6, B9, B12, Fe, and Zn. Fortification levels were adjusted to reflect estimated compliance and micronutrient degradation, ensuring realistic nutrient profiles for fortified foods at the household level [19]. In addition, supplement intake was not captured in the IHS5 and consequently could not be incorporated into apparent micronutrient intake estimates.

### Data analysis

We estimated the apparent daily intake per AFE for 15 micronutrients comprising vitamins A (RAE), C, E, B1, B2, B3, B6, B9, and B12, and the minerals Ca, Cu, Fe, Mg, Se, and Zn. These micronutrients were selected because reliable food composition data were available from the 2019 Malawian FCT, ensuring more reliable nutrient matching, and because corresponding Harmonized Average Requirement (H-AR) values were available for assessing adequacy [35]. For Fe, we utilized the Institute of Medicine full probability approach, adjusted for a 5% iron bioavailability, consistent with predominantly cereal-based diets commonly consumed in Malawi [7]. For Zn, we assumed a low bioavailability because Malawian diets are characterized by high intakes of unrefined grains and legumes with high phytate content, which reduces Zn bioavailability [7]. For Ca, phytate/oxalate can reduce absorption, but given uncertainty about effect size across food matrices and the absence of widely adopted adjustment guidelines for population‑level assessments, we did not apply a separate bioavailability correction [36, 37]. Apparent intake was estimated as the amount of food consumed per day per AFE as represented by the following equation:

$$\:{Apparent\;intake\;left}\:\left(\frac{g}{d}/AFE\right)=\left(\frac{Quantity\:of\:\mathrm{f}\mathrm{o}\mathrm{o}\mathrm{d}\:consumed\:by\:a\:household\:\left(\frac{g}{d}\right)\:}{{\sum\:}_{AFE}\:}\right)$$

where AFE = the number of AFEs in a household

The estimated micronutrient intakes were compared against H-AR values for a non-pregnant, non-lactating, adult female aged 18–29 years to categorize households with low apparent intakes per day per AFE for the 15 micronutrients, represented by the following equation:

$$\:\left(\frac{Daily\:household\:micronutrient\:supply\:\left(units\right)\:\:}{{\sum\:}_{AFE}}\right)<{H-AR}_{adult\:females}$$

To calculate the prevalence of inadequate apparent intake, those households categorized with low apparent intakes per day per AFE were divided by the number of households in each specific subpopulation group.

**Fig. 1:**
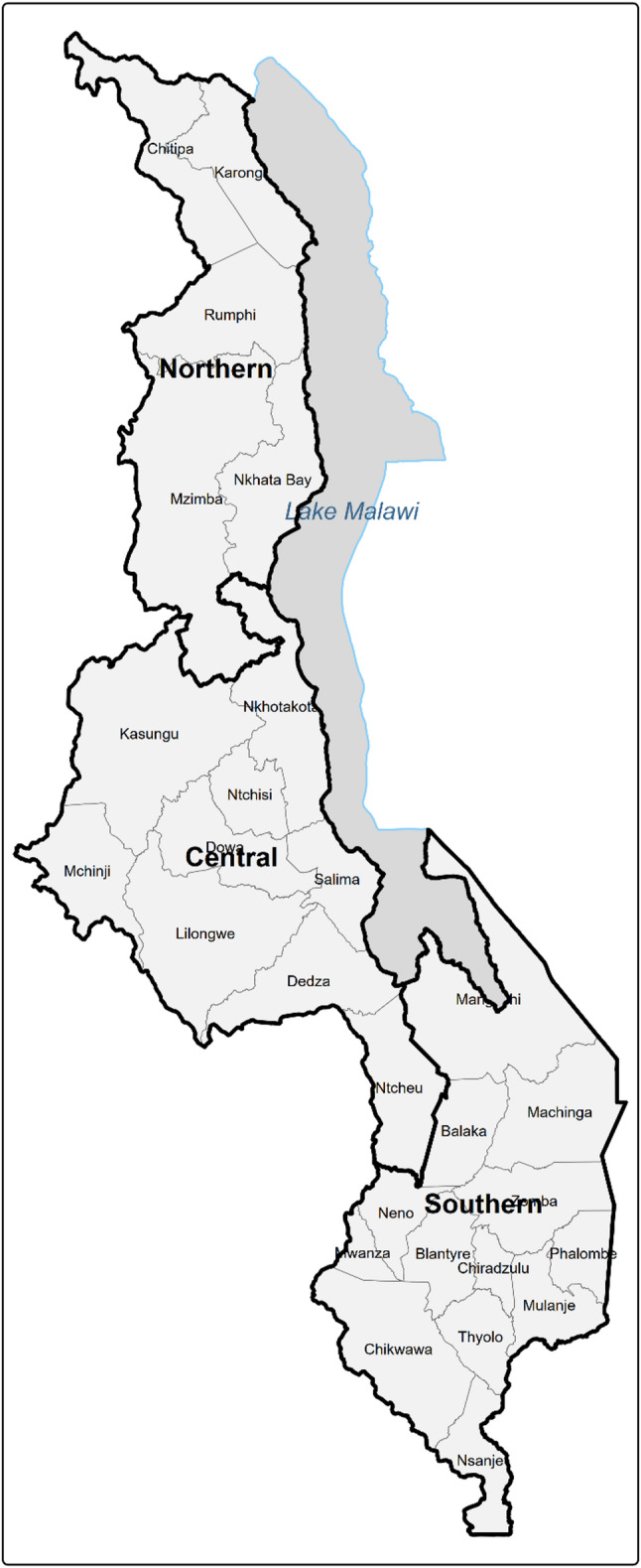
Administrative regions and districts of Malawi

To identify micronutrients of public health concern, we focused on those with a national prevalence of inadequate apparent intake of ≥ 20%. This threshold aligns with international guidance, which considers the prevalence of deficiency of 20% or more as indicative of a public health problem warranting intervention. For example, the World Health Organization (WHO) classifies a prevalence of low serum Zn concentrations above 20% as a public health concern requiring action [38]. Therefore, applying this threshold provides a consistent and evidence-based approach for identifying nutrients of concern in population-level dietary assessments. For micronutrients meeting this criterion, we further estimated prevalence of inadequate apparent intake per day per AFE stratified by region, residence and socioeconomic quintiles, identified major dietary sources of each micronutrient, and assessed geographic variation in the prevalence of inadequate apparent intake at the district level. District-level aggregation was based on Malawi’s administrative boundaries obtained from the NSO. These shapefiles were used for spatial aggregation and visualization. For clarity and orientation, an administrative map showing Malawi’s districts and the three administrative regions (Northern, Central, and Southern), which form the basis for geographic stratification and spatial analysis in this study, is provided in Fig. 1.

Results were reported at national, regional and districts levels, and further stratified by urban versus rural households and by socioeconomic quintiles. Socioeconomic quintiles were determined using total inflation-adjusted annual household expenditure per capita, as provided in the IHS5 dataset. All data cleaning, transformation, and analysis were conducted in R (version 4.1.3; R Foundation for Statistical Computing). Survey weights from IHS5 were applied using functions from the *srvyr* package. Prevalence estimates were calculated as weighted proportions using survey weights, and 95% confidence intervals (95% CI) were obtained from design-adjusted variance estimates to account for the complex sampling design for the district-level estimates. For spatial analysis, we used the *sf* and *tmap* packages, and visualizations were created with *ggplot2*.

## Results

The IHS5 collected food consumption data from 11,432 households, of which 9342 (82%) were rural and 2090 (18%) were urban. Within each group, households were classified into five socioeconomic quintiles based on total inflation-adjusted per capita annual household expenditure, with an almost equal number of households in each quintile. Table 1 summarizes the characteristics of the sampled households. Compared to urban households, rural households were less likely to be headed by a man, and their household heads had lower education levels, and were slightly older on average. Rural households were also larger in size than urban households, located approximately four times farther from main agricultural markets, and roughly 10 km from the main road.

**Table 1:** Household descriptive summary from the 2019/2020 Malawi’s Fifth Integrated Household Survey

Residence	Rural					Urban					
Socioeconomic position by quintile of total annual household expenditure	Lowest	Lower middle	Middle	Upper middle	Highest	Lowest	Lower middle	Middle	Upper middle	Highest	*P*-value (urban/rural)^a^
Households (*n*)	1869	1869	1868	1868	1868	418	419	418	417	418	
Household headship (%)											< 0.001
Male	65	69	67	69	72	77	77	79	76	77	
Female	35	31	33	31	28	23	23	21	24	23	
Highest education attainment of household head (%)											< 0.001
No education	85	76	73	66	49	56	42	29	21	12	
Primary	8.8	12	13	14	15	20	16	18	16	5.6	
Secondary	6.5	11	15	19	31	23	40	49	49	42	
More than secondary	1	0.3	0.2	0.6	5.2	0.8	2.0	3.0	14	40	
Mean age of household head (years)	44	43	44	44	44	41	39	39	40	39	< 0.001
Mean size of households	5.7	5.0	4.6	4.0	3.0	5.6	4.7	4.3	3.7	2.9	< 0.001
Mean distance (km) to the nearest:											
Major road	11.2	10.8	10.3	9.4	8.8	1.7	1.8	1.9	1.8	1.8	< 0.001
Agricultural market	24.8	24.6	24.5	24.1	25.0	7.2	7.1	6.1	6.4	6.2	< 0.001

^a^Urban/rural differences within variables tested using Pearson’s Chi-squared test for categorical variables and independent t-test for continuous variables (age of household head, household size, and distance)

### Prevalence of apparent micronutrient inadequacies

**Fig. 2:**
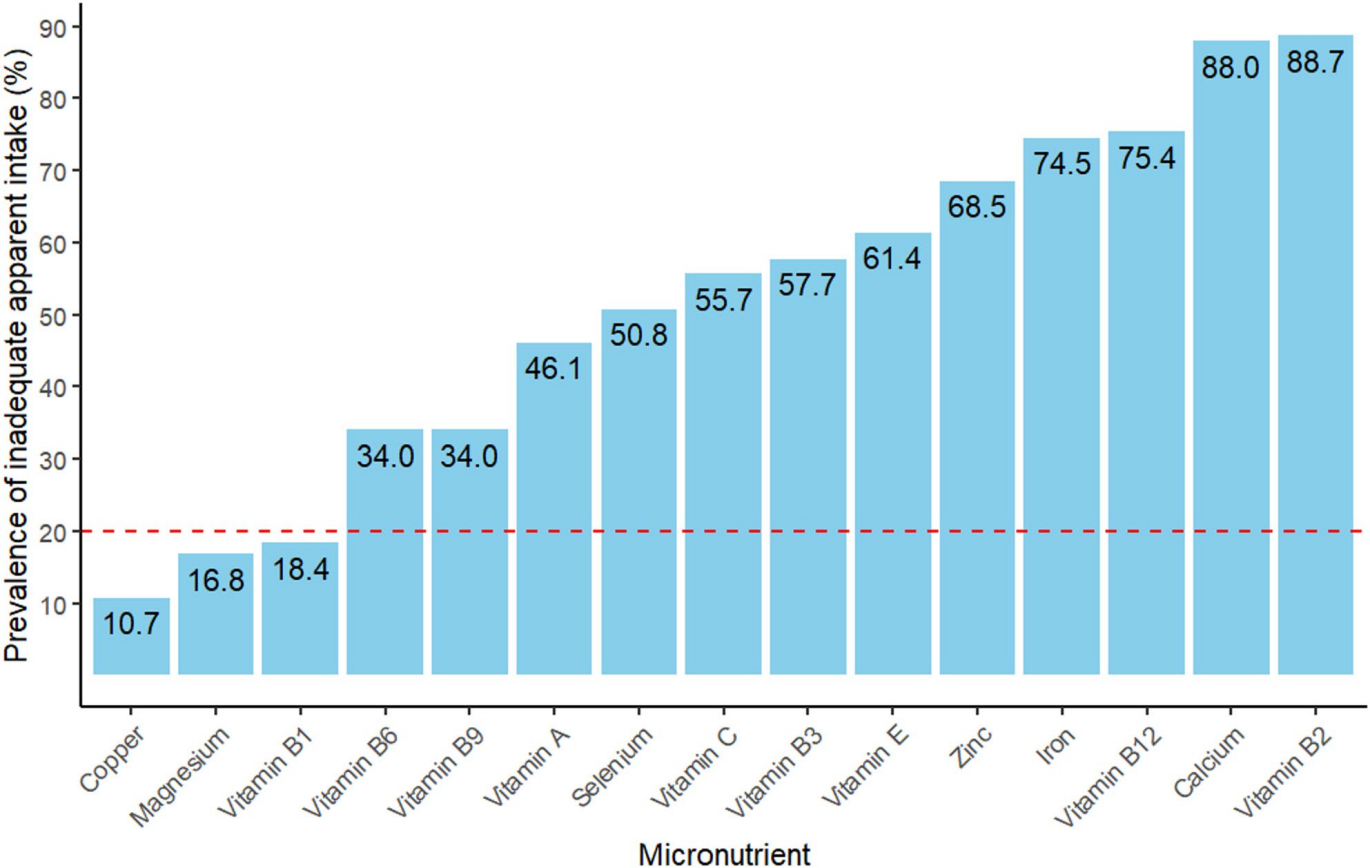
Percentage of inadequate apparent intakes for 15 micronutrients, Malawi (*n* = 11432 households)

Figure 2 presents the percentage of inadequate apparent intakes for 15 micronutrients. Nationally, inadequacy exceeded 20% (red dashed line) for 12 micronutrients: vitamins A, B2, B3, B6, B9, B12, C, and E, and the minerals Ca, Fe, Se, and Zn. Among these micronutrients, vitamin B2 (88.7%) and Ca (88.0%) showed the highest prevalence of inadequacy, followed by vitamin B12 (75.4%) and Fe (74.5%).

### Prevalence of inadequate apparent intakes of selected vitamins

Table 2 shows the prevalence of inadequate apparent intakes for eight vitamins disaggregated by region, residence, and socioeconomic position (SEP). The differences by SEP were particularly pronounced, with the lowest quintiles facing very high inadequacy for nearly all vitamins, while the highest quintiles had substantially lower prevalence. Although inadequacy declined with higher SEP and urban residence, some vitamins, such as vitamin B2 and B12 remained problematic even among better-off and urban households. Regional variation was evident but less striking than socioeconomic differences.

**Table 2:** Percentage of inadequate apparent intakes for vitamins A, C, E, B2, B3, B6, B9, and B12, disaggregated by region, residence, and socioeconomic position

Population	Households (*n*)	Vit. A – RAE	Vit. C	Vit E	Vit. B2	Vit. B3	Vit. B6	Vit. B9	Vit. B12
		Percentage (%)							
National (total)	11,432	46.1	55.7	61.4	88.7	57.7	34.0	34.0	75.4
*Administrative region*									
Northern region	2176	37.9	54.4	60.3	86.1	54.5	36.7	32.5	64.6
Central region	3951	49.4	60.9	66.7	92.5	64.2	42.9	41.4	84.2
Southern region	5305	45.5	51.2	56.7	86.0	52.6	25.1	27.6	70.3
*Residence and SEP by quintile of total annual household expenditure per capita*									
Rural	9342	50.7	56.4	63.9	90.3	60.1	35.4	36.8	79.6
Lowest	1869	82.4	80.3	94.6	99.6	94.9	68.5	74.0	96.5
Lower middle	1869	66.4	66.9	81.9	98.9	81.4	45.9	50.5	91.9
Middle	1868	52.0	57.2	66.1	96.8	60.3	29.2	29.3	84.9
Higher middle	1868	33.7	45.0	48.2	90.2	40.4	18.8	16.9	73.2
Highest	1868	10.5	26.3	20.4	61.8	14.4	7.7	5.5	45.8
Urban	2090	22.8	52.2	48.4	80.8	45.1	27.1	19.8	54.1
Lowest	418	64.4	83.1	85.8	99.4	93.6	59.6	56.4	86.5
Lower middle	419	25.3	63.1	65.6	97.8	64.9	38.3	21.3	67.9
Middle	418	11.4	48.2	46.1	89.6	37.2	21.2	9.6	54.6
Upper middle	417	4.4	36.9	22.8	72.8	14.0	6.7	4.7	35.0
Highest	418	1.9	22.7	12.8	37.6	5.4	2.8	0.7	18.6

### Prevalence of inadequate apparent intakes of selected minerals

Table 3 shows the prevalence of inadequate apparent intakes for four minerals disaggregated by region, residence, and SEP. Inadequacy was widespread for all minerals, but the extent varied considerably across demographic groups. The burden was disproportionately concentrated among poorer households, with the lowest SEP quintiles exhibiting high inadequacy for all four minerals. Although inadequacy declined with higher SEP and urban residence, Ca and Fe remained problematic even among better-off households. Regional differences were evident, with the central region generally showing higher inadequacy.

**Table 3:** Percentage of inadequate apparent intakes for minerals (Ca, Fe, Se, and Zn), disaggregated by region, residence, and socioeconomic position

Population	Households (*n*)	Ca	Fe	Se	Zn
		Percentage (%)			
National (total)	11,432	88.0	74.5	50.8	68.5
*Administrative region*					
Northern region	2176	79.0	74.6	45.4	69.0
Central region	3951	93.5	81.9	61.8	77.0
Southern region	5305	85.5	67.6	42.2	60.5
*Residence and SEP by quintile of total annual household expenditure per capita*					
Rural	9342	88.8	74.5	52.4	69.0
Lowest	1869	97.5	92.8	90.9	96.0
Lower middle	1869	96.2	84.4	70.9	84.8
Middle	1868	93.7	75.6	50.3	71.6
Higher middle	1868	88.5	66.4	30.2	54.9
Highest	1868	64.8	48.2	10.7	30.0
Urban	2090	83.7	74.7	42.5	66.0
Lowest	418	97.7	92.0	85.2	93.9
Lower middle	419	94.5	82.9	57.4	79.3
Middle	418	89.6	77.9	35.9	68.9
Upper middle	417	78.3	68.4	15.7	52.3
Highest	418	53.7	47.7	9.2	28.3

### Food group contributions to selected micronutrients

**Fig. 3:**
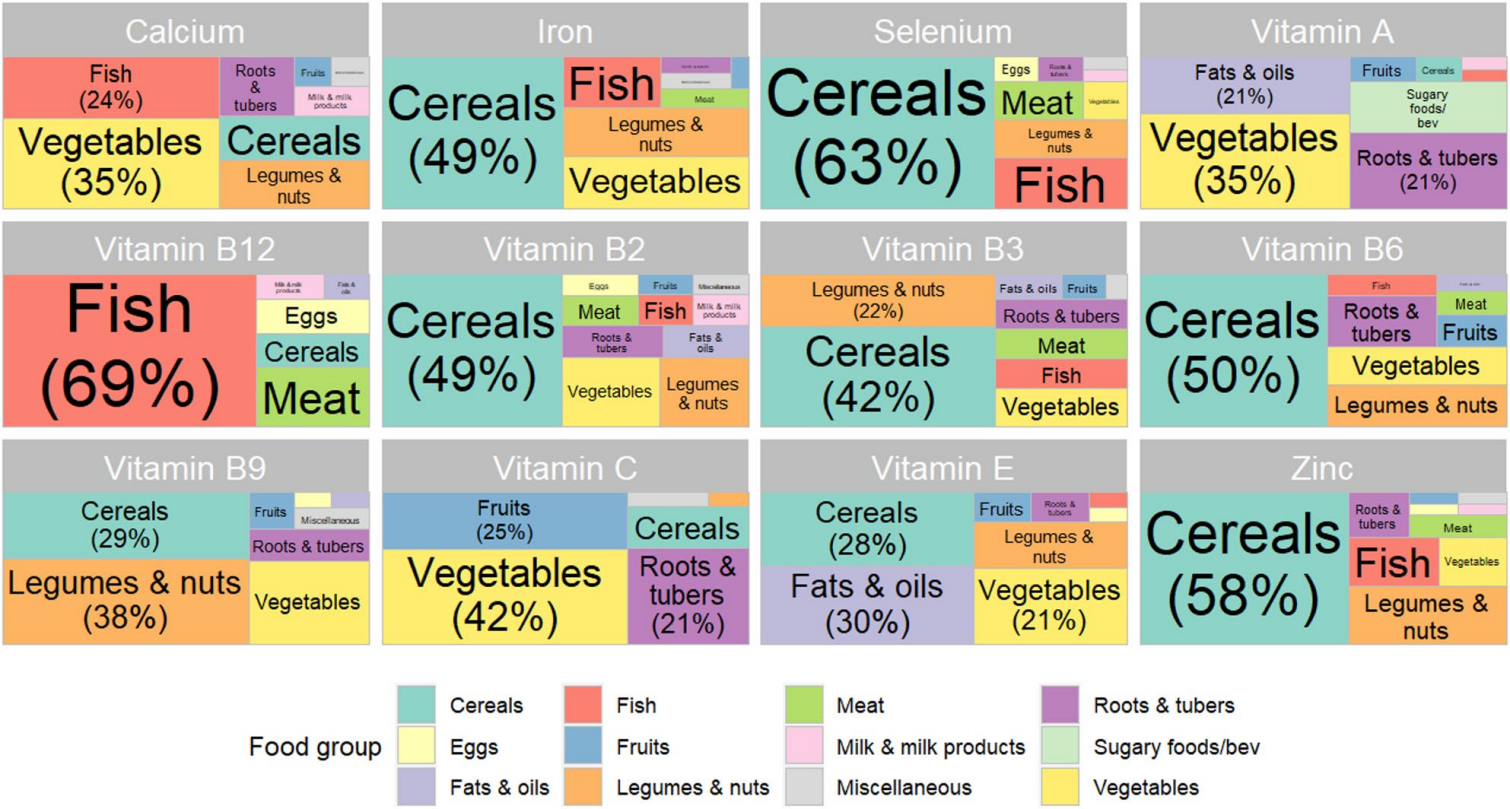
Proportion of food groups per day per AFE supplying apparent intake of 12 selected micronutrients

Figure 3 illustrates the contribution of different food groups to apparent intake of the 12 micronutrients with a national prevalence of inadequacy above 20%. Cereals were the dominant source for several nutrients, supplying nearly half or more of the apparent intake for vitamins B2 (49%), B3 (42%), and B6 (50%), as well as Fe (49%), Se (63%), and Zn (58%). Fish were the primary source of vitamin B12, while vegetables contributed most to vitamins A and C, and Ca apparent intakes. Vitamin E apparent intake was distributed across multiple food groups, with notable contributions from fats and oils, cereals, and vegetables. Additional Fig. 1 presents a comparison of food group contributions between rural and urban households. The overall patterns were similar to national trends, but rural diets relied more heavily on cereals for several micronutrients.

### Geographic distribution of inadequate micronutrient apparent intake

**Fig. 4:**
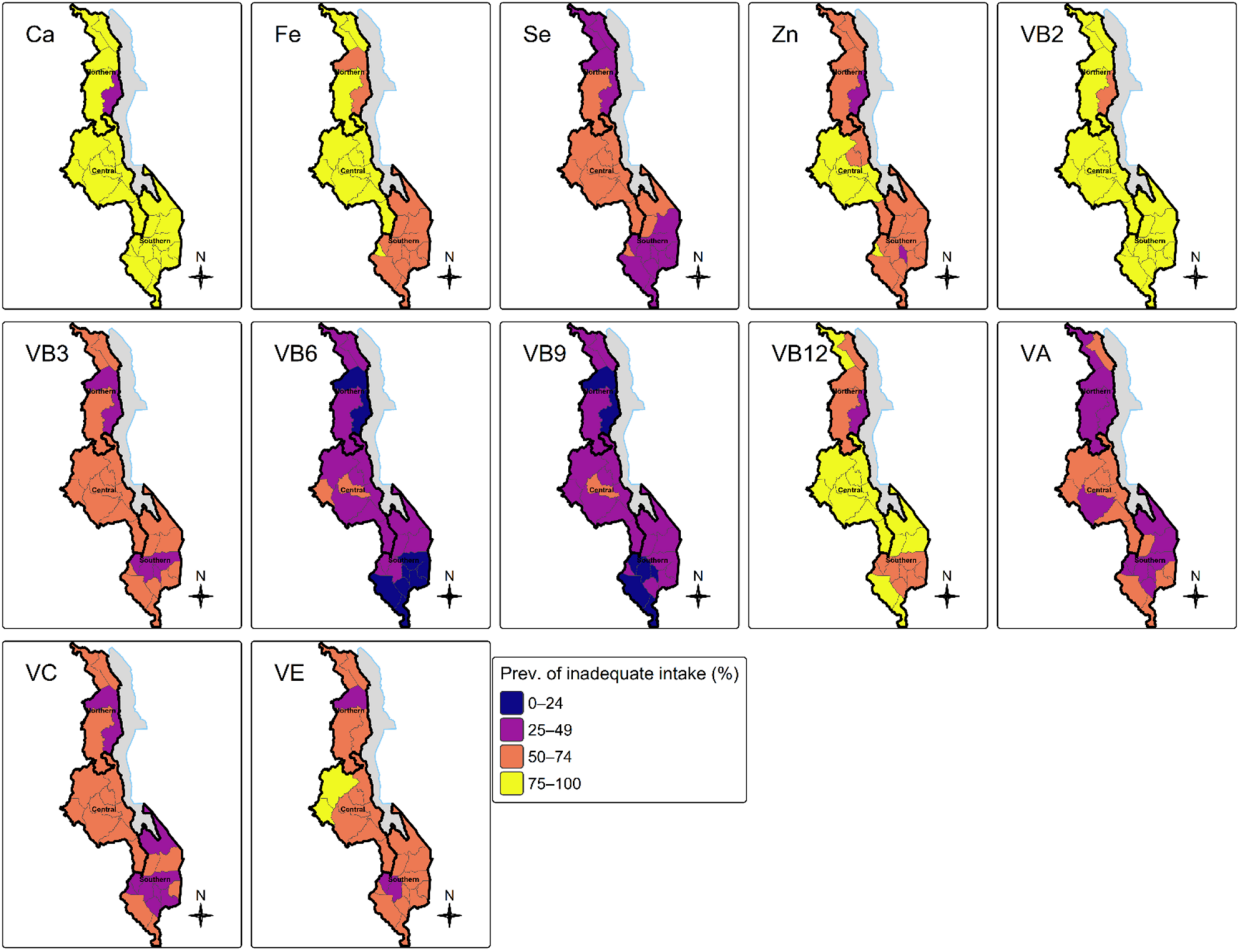
Prevalence of inadequate apparent intake for the 12 selected micronutrients by district Key: Ca=Calcium, Fe=Iron, Se=Selenium, Zn=Zinc, VB2 = vitamin B2, VB3 = vitamin B3, VB6 = vitamin B6, VB9 = vitamin B9, VB12 = vitamin B12, VA=vitamin A (RAE), VC=vitamin C, VE=vitamin E

Figure 4 displays district-level prevalence of inadequate apparent intake for the 12 micronutrients with national inadequacy above 20%. The maps reveal substantial geographic heterogeneity across Malawi. Districts in the central region consistently showed higher prevalences for most micronutrients compared to those in the northern and southern regions, although pockets of high inadequacy were evident elsewhere. Vitamin B2 and Ca inadequacies were particularly widespread, with many districts showing prevalences exceeding 75%. The prevalence of apparent inadequate intake for Fe and Zn was also high, especially in central and selected southern region districts. For vitamins A, C, and E, geographic variation was evident, with clusters of high prevalence observed in central and parts of the southern region. Other B-vitamins (B3, B6, B9, and B12) and Se exhibited moderate to high inadequacy with notable differences across districts and regions. Detailed district-level estimates, including point prevalence and 95% CI, are provided in Additional Table 2 and Additional Table 3. Note that median daily energy apparent intake per AFE varied across regions, with higher values observed in urban areas and in the southern and northern regions compared to rural areas and the central region. Detailed energy apparent intake distributions by residence and district are provided in Additional Table 4.

## Discussion

### Overview of micronutrient inadequacies

In this study, we estimated the prevalence of risk for inadequate apparent intake of 15 micronutrients at the national level and further disaggregated by place of residence (rural vs. urban), socioeconomic quintile, region, and district using HCES data. We found a high prevalence of inadequate apparent intake for 12 micronutrients – vitamins A (RAE), C, E, B2, B3, B6, B9, B12, and the minerals Ca, Fe, Se, and Zn exceeding the 20% inadequacy threshold nationally. These findings are consistent with anticipated patterns for certain nutrients such as vitamin B2, B12, Ca, and Zn, whose primary sources are animal-based foods (e.g., meat, fish, dairy, and eggs). These foods are not widely consumed in Malawi, particularly among rural households and those in the lowest socioeconomic groups [7, 20]. Limited access to and affordability of animal-source foods, combined with cultural dietary patterns that emphasize maize as the primary staple, are well-documented contributors to inadequate diet quality in Malawi, affecting both macronutrient balance (e.g. protein quality) and micronutrient intake [39, 40].

The findings of this study partly align with the 2015/16 MNS, which assessed micronutrient status using biomarkers – reflecting absorption, metabolism, and physiological regulation, whereas our estimates capture dietary exposure [41, 42]. Notably, the survey was conducted during the El Niño‑related 2015–2016 drought, a period associated with short‑term deterioration in diet quality and food security, which is important to consider when interpreting biomarker–diet comparisons [11, 12]. These climate shocks disrupted food availability and markets and could plausibly elevate the deficiency for some micronutrients such as Zn and vitamin B12. The 2015/16 MNS reported widespread deficiencies (e.g. Zn: 60–69% across groups; Fe deficiency: 22% in preschool children and 15% in women of reproductive age [WRA]; vitamin B12 depletion in 40.5% of non‑pregnant WRA). In addition, national Se analyses reported low plasma Se in 62.5% of WRA and 57.2% of men, consistent with the high prevalence of inadequate Se intake in our study [43]. While agreement is strong for some micronutrients, notable differences remain for vitamin A: the MNS reported very low deficiency (vitamin A < 4% across groups), whereas our estimates suggest higher inadequacy (46.1% vitamin A). These gaps likely reflect the seasonal timing of the MNS (December – February), overlapping with periods of higher carotenoid‑rich fruit intake (e.g. mango) that can elevate vitamin A status, and potential under‑capture of some vitamin A‑rich foods in the IHS5 food list (e.g. vitamin A maize). These considerations reinforce the importance of triangulating dietary apparent intake estimates (which identify contributing foods and overall diet composition and patterns) with biomarker evidence and contextual factors such as seasonality and supplementation program coverage when assessing micronutrient adequacy.

### Geographic and socioeconomic disparities

Our findings also revealed geographic and socioeconomic disparities in apparent inadequate micronutrient intakes across Malawi. While apparent inadequate micronutrient intakes are a national challenge, affecting all regions, districts in the central region consistently showed higher prevalence for several micronutrients (e.g. Fe, vitamin B12) compared to those in the northern and southern regions. Poverty likely exacerbates these risks. According to the Malawi Poverty Report 2020, central region households experienced the highest poverty (55.8%) and ultra-poverty (25.4%), compared to southern (51.0% and 19.1%) and northern (32.9% and 8.6%) regions [22]. Poor households consumed, on average, about 17% below the poverty line, with rural areas, including much of central region – showing deeper shortfalls. This economic vulnerability translates into limited dietary diversity and reduced access to nutrient-rich foods such as fish and dairy products. Recent evidence underscores this challenge: Dedza district (central region) recorded the lowest mean dietary diversity scores and the highest proportion of women (89%) and children (88.5%) failing to meet minimum dietary diversity, compared to Balaka (southern) and Mzimba (northern) [44].

Furthermore, although urban households typically live closer to markets and have higher educational attainment, our analysis still showed a high prevalence of apparent micronutrient inadequacy in urban settings. This likely reflects higher food prices and diet costs that limit purchase of nutrient‑dense foods, alongside dietary patterns that are energy‑adequate but micronutrient‑poor. Importantly, urban areas are heterogeneous: inadequacy decreased with socioeconomic status within urban settings (e.g. peri‑urban vs. wealthier areas), indicating that affordability and purchasing power remain decisive determinants even where physical access to markets is better [45].

The present findings show both alignment and divergence with the 2015/16 MNS across regions and socioeconomic groups. For vitamin B12, the MNS reported the highest deficiency among WRA in the central region (18.8%), followed by the northern (12.8%) and southern (7.6%) regions – patterns that correspond with our apparent intake estimates (Table 2). In contrast, while the MNS reported the highest Zn deficiency in the southern region (67.0%), our analysis indicated the highest apparent inadequacy in the central region (77.0%) (Table 3); however, both datasets consistently show a high regional burden of Zn deficiency and inadequacy. Socioeconomically, apparent intake-based estimates display a clear gradient, with poorer households showing higher inadequacy, whereas MNS biomarkers present more variable SEP patterns; some (e.g. vitamin B12) higher among lower‑income groups, others (e.g. Zn and Fe) uniformly high across SEP. These results underscore the need for both universal and region-and even district-specific strategies to promote dietary diversification and improve access to nutrient-rich foods. Malawi’s decentralized governance system enables each of the 28 districts to plan and implement their own development initiatives, allowing for tailored approaches [46]. In the short term, these findings can be used to prioritize a limited set of high‑burden micronutrients, particularly vitamin B2, Ca, Fe, Se, and Zn for programmatic attention, rather than attempting to address all micronutrients simultaneously. The results can also inform refinement of existing LSFF and biofortification programmes, including assessment of whether current vehicles and premix formulations adequately address observed dietary gaps. National interventions such as agronomic biofortification of maize using Se-enriched fertilizers are critical given maize’s central role in the diet [47]. At the same time, district-level strategies should address local dietary gaps, for example by promoting fish and legume consumption and improving affordability of nutrient-dense foods through poverty reduction initiatives and social protection programs. Future research should integrate dietary intake, biomarker data, and socioeconomic indicators to refine targeting and monitor the impact of interventions.

### Neglected nutrients and utility of HCES data

The 2015/16 Malawi MNS assessed only a limited set of nutrients – vitamins A, B9, B12, Fe, iodine, and Zn, using biomarkers. While biomarkers offer robust and objective measures of nutritional status, their application is often constrained by high costs, logistical complexity, and the need for specialized equipment and trained personnel [48]. These challenges contribute to the narrow scope of micronutrients typically included in national surveys. As a result, it becomes difficult to assess and compare the status of other critical micronutrients such as vitamin B2, B3, and Se, which are not routinely measured. Given the widespread dietary apparent inadequate intakes indicated by this study, we recommend that future national micronutrient surveys expand their scope to include some of these “neglected micronutrients.” Doing so would allow for a more comprehensive understanding of population-level micronutrient deficiencies across demographic groups and help validate whether dietary apparent inadequacies are reflected in biochemical status. In the near term, a practical and cost-efficient approach would be to implement a phased expansion of biomarker coverage, beginning with small sentinel studies (e.g. every 2–3 years) focused on micronutrients such as vitamin B2 and Se. Such a targeted expansion would substantially strengthen the evidence base for nutrition programming in Malawi.

Greater attention to these ‘neglected’ micronutrients is warranted because they play essential roles in metabolic, neurological, immune, and antioxidant functions. Although our study does not assess clinical outcomes, inadequate intakes of micronutrients such as vitamin B2, B3, E, Ca, and Se are associated with impaired physiological functioning and increased vulnerability to poor health [49–57].

Although we recommend expanding future micronutrient surveys to include these “neglected micronutrients,” we acknowledge that reliable biomarkers for some micronutrients such as Ca are not available or widely agreed upon [3]. This limitation underscores the value of dietary data from sources such as HCES as a starting point for identifying potential gaps in population diets and prioritizing interventions, even in the absence of biomarker-based deficiency estimates. HCES offer several advantages: they are conducted regularly (every 3–5 years), are nationally representative, and use standardized methods, enabling the examination of trends over time and comparisons across regions and socioeconomic groups. Malawi has implemented six rounds of HCES between 1997 and 2024, providing a rich and recurrent data source from which trends in inadequate apparent micronutrient intakes can be assessed. Because full national biomarker-based micronutrient surveys are far more resource intensive and generally feasible only every 5–10 years, HCES can provide valuable ‘in between’ data to monitor dietary exposure and identify priority micronutrients requiring attention, as demonstrated in this study using the most recent IHS5 dataset. While dietary intake data cannot replace biomarkers, combining them with targeted biomarker measurements where feasible would generate stronger evidence base for policy and programme design. In the interim, HCES findings can be used to prioritize at-risk nutrients and population groups for immediate action and guide small sentinel biomarker assessments to provide timely updates between national survey rounds. Beyond policy planning, these findings can be usable in routine services: practitioners can prioritize counselling on the most at-risk micronutrients and deliver simple food-based recommendations using locally available foods.

### Study strengths and limitations

Food consumption data from HCES, such as IHS5, are an essential source of information on food security and nutrition in Malawi. One of the major strengths of this study is the use of HCES data, which is collected every 3–5 years using standardized methods and collects nationally representative data. The survey covered a large sample across all regions of Malawi, including urban and rural areas, enabling robust disaggregation and comparison of inadequate apparent micronutrient intakes across geographic and socioeconomic groups. This consistency and breadth make HCES a reliable and rich source of information on household food consumption and socio-demographic characteristics. In addition, most micronutrient values (71%) came from the Malawian FCT, which best reflects locally consumed foods. Where local values were missing, we used regional FCTs; these primarily contributed nutrients such as Se and vitamin E for items like selected fish and some processed foods. This supplementation ensured complete coverage while prioritizing local data, although minor under‑ or over‑estimation is possible where regional entries may not perfectly match Malawian varieties.

However, we acknowledge the following limitations. First, HCES collects food consumption data at the household level, which assumes equitable intra-household food distribution when calculating the AFE. This assumption may not hold true, especially in households with diverse age and gender compositions. Second, the HCES uses a 7-day recall based on predetermined list of food items (*n* = 135 foods) which is neither a comprehensive list of all foods consumed in Malawi, nor does it always provide a detailed description of these foods to precisely match all foods to appropriate food composition tables, particularly for the fish [18]. Third, the HCES does not capture information on cooking methods or preparation practices, and certain micronutrients, especially heat- and water-sensitive B-vitamins and vitamin C can degrade during cooking; because such detail is absent, some misestimation of apparent micronutrient intake is possible. Fourth, the IHS5 did not capture food consumed outside the home, which can be a significant portion of dietary intake, especially in urban settings. This omission may lead to underestimation of total energy and nutrient intake.

Finally, the overall median daily apparent energy intake per AFE was substantially lower in central region (1725 kcal; Interquartile range [IQR]: 1205–2502) compared to the northern region (2130 kcal; IQR: 1588–2957) and southern region (2080 kcal; IQR: 1464–2925). Lower energy apparent intake likely translates into lower absolute micronutrient intakes, thereby amplifying inadequacy estimates. Such discrepancies may stem from enumerator variability across districts, which can influence adequacy estimates, particularly for micronutrients derived from energy-dense staples (e.g. B vitamins, Se, and Zn from cereals) compared to those obtained from low-energy, micronutrient-dense foods such as fruits and vegetables (e.g., vitamin C). Consistent with IHS5 context variables, districts showing higher apparent inadequacy also tended to have lower per‑capita energy availability and a greater reliance on staple cereals, particularly among rural households. This suggests that affordability constraints and diet composition rather than enumerator effects alone likely underlie part of the observed disparities. To build on these descriptive patterns, future work could apply multivariable regression using IHS5 microdata to examine how household characteristics, market access, and seasonality independently contribute to inadequate apparent intake. Although some districts showed low apparent energy values, reflecting differential errors in estimating food intakes, the estimated energy apparent intakes for most districts appeared broadly reasonable when compared to the expected requirements for non-pregnant, non-lactating WRA (Additional Table 4). Hence, HCES offers a feasible and informative approach to understanding population-level nutritional risks in Malawi, complementing biomarker-based assessments and supporting timely, evidence-based decision-making [18].

## Conclusion

This study provides evidence that the risk of apparent dietary multiple micronutrient inadequacies is potentially widespread and geographically varied across Malawi. Using nationally representative HCES data, we identified a high prevalence of inadequate apparent intakes for 12 micronutrients, including vitamins A, C, E, B2, B3, B6, B9, B12, and minerals Ca, Fe, Se, and Zn. These apparent inadequacies are particularly pronounced in the central region districts and among rural and low-income households. In the short term, these findings can be used to prioritize a limited set of high‑burden micronutrients, particularly vitamin B2, Ca, Fe, Se, and Zn, for programmatic attention within existing nutrition policies. Addressing these challenges will require district-specific and socioeconomically tailored strategies that promote dietary diversification and improve access to diverse, nutrient-dense foods. Integrating apparent intake data with biomarker assessments is essential for generating a more comprehensive and actionable evidence base to guide effective micronutrient policies and programs in Malawi. While the findings are most directly relevant for Malawi, the analytical approach demonstrates how routinely collected HCES data can be used to identify priority micronutrients and geographic disparities in other resource-constrained settings.

## Supplementary Information

Additional file 1: Tables 1a and 1b: Example of household Adult Female Equivalent (AFE) allocation and apparent intake calculation.

Additional file 2: Figure 1. Proportion of food groups per day per adult female equivalent supplying apparent intake of 12 selected micronutrients stratified by residence (rural vs urban).

Additional file 3: Table 2. District-level prevalence of inadequate apparent intakes for vitamins A, C, E, B2, B3, B6, B9, and B12, with 95% confidence intervals.

Additional file 4: Table 3: District-level prevalence of inadequate apparent intake for minerals (Ca, Fe, Se, and Zn), with 95% confidence intervals.

Additional file 5: Table 4: Median apparent energy intake per day per adult female equivalent disaggregated by residence and district.

## Data Availability

The dataset (The 2019/20 Malawi’s Fifth Integrated Household Survey [IHS5]) generated and/or analysed during the current study are available in the World Bank repository at [https://microdata.worldbank.org/index.php/catalog/3818 ]. The source code for cleaning and preprocessing the IHS5 food consumption data is publicly available in a GitHub repository at [https://github.com/Gare94/ihs5_cleaning_github https://github.com/Gare94/ihs5_cleaning_github ].

## Abbreviations

AFE: Adult Female Equivalent
FCT: Food Composition Table
HCES: Household Consumption and Expenditure Survey
H-AR: Harmonized Average Requirement
IHS: Integrated Household Survey
IQR: Interquartile range
LSFF: Large-Scale Food Fortification
MDHS: Malawi Demographic Health Survey
NSO: National Statistical Office
PAL: Physical Activity Level
SEP: Socioeconomic Position
WRA: Women of Reproductive Age
