# Supplementary material for: The risk of dietary multiple micronutrient inadequacies is widespread and geographically varied in Malawi
Source: BMC Nutr. 2026 May 25;12:147. doi: 10.1186/s40795-026-01369-2 (PMC13412303; doi:10.1186/s40795-026-01369-2)
Supplement: Supplementary file 1 — Additional file 1: Tables 1a and 1b: Example of household Adult Female Equivalent (AFE) allocation and apparent intake calculation. [file 40795_2026_1369_MOESM1_ESM.docx]

**Additional file 1**

**Additional Table 1a:** Example of household Adult Female Equivalent AFE allocation

| **Household Member** | **Age (years)** | **Energy Requirement (kcal/d)** | **AFE** |
| --- | --- | --- | --- |
| 1. Man | 31 | 2850 | 1.24 |
| 1. Woman | 26 | 2300 | 1.0 |
| 1. Child 1 (F) | 9 | 1850 | 0.80 |
| 1. Child 2 (M) | 4 | 1350 | 0.59 |
| **Total household AFE** | | | **3.63** |

This worked example demonstrates how Adult Female Equivalents (AFEs) are calculated and used to allocate individuals proportionally to their energy needs (**Additional Table 1a**). Each household member is assigned an AFE value based on their age‑, sex‑, and physiological‑status‑specific energy requirements, relative to the reference requirement of a non‑pregnant, non‑lactating woman aged 18–29 years. The sum of all AFEs in the household represents the total energy‑demand units used to derive apparent intake for the reference individual.

**Additional Table 1b:** Example of converting household food quantity into apparent intake for the reference adult female

| **Step** | **Description** | **Value** | **Calculation** |
| --- | --- | --- | --- |
| 1 | Weekly quantity | 700 g | Reported in HCES |
| 2 | Daily quantity | 100 g | 700/7 |
| 3 | Total Household AFE | 3.63 | From Table X |
| 4 | Apparent intake per AFE | 27.5 g | 100/3.63 |
| 5 | Reference woman apparent intake | 27.5 g | = Step 4 |

**Note:** This example uses the same four‑member household from **Additional Table 1a**
(Total Household AFE = **3.63**). The household reports consuming **700 g of maize flour over 7 days**

**Additional Table 1b** illustrates how household‑level food quantities are converted into apparent intake for the reference individual. In this example, a household reports consuming 700 g of maize flour in 7 days. The weekly quantity is converted into a daily amount and then divided by the total household Adult Female Equivalents (AFE = 3.63). The resulting value (26.5 g/day) represents the apparent daily maize flour intake for a non‑pregnant, non‑lactating woman aged 18–29 years. This demonstrates how proportional AFE allocation links household food availability to individual‑level apparent intake estimates.
