# Supplementary material for: The risk of dietary multiple micronutrient inadequacies is widespread and geographically varied in Malawi
Source: BMC Nutr. 2026 May 25;12:147. doi: 10.1186/s40795-026-01369-2 (PMC13412303; doi:10.1186/s40795-026-01369-2)
Supplement: Supplementary file 2 — Additional file 2: Figure 1. Proportion of food groups per day per adult female equivalent supplying apparent intake of 12 selected micronutrients stratified by residence (rural vs urban). [file 40795_2026_1369_MOESM2_ESM.docx]

**Additional file 2**

**Additional Figure 1:** Proportion of food groups per day per adult female equivalent supplying apparent intake of 12 selected micronutrients stratified by residence (rural vs urban)


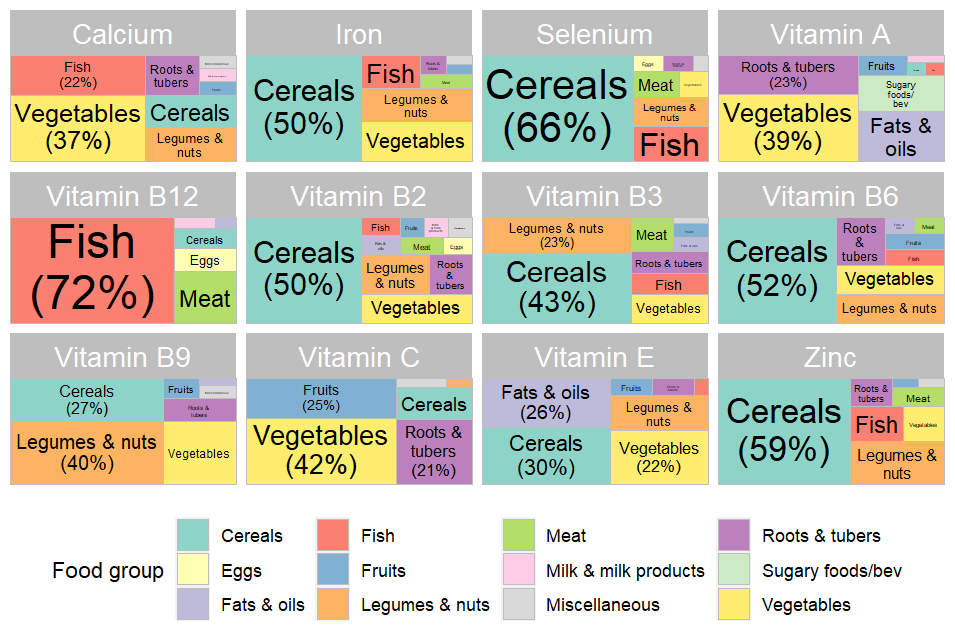

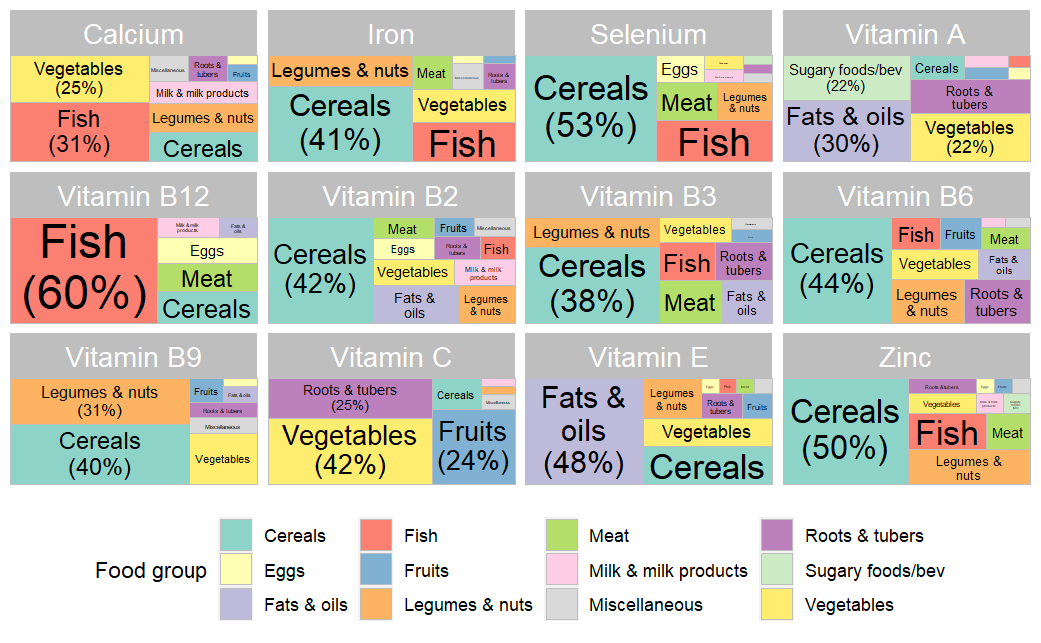


**Rural**

**Urban**
