# Supplementary material for: The risk of dietary multiple micronutrient inadequacies is widespread and geographically varied in Malawi
Source: BMC Nutr. 2026 May 25;12:147. doi: 10.1186/s40795-026-01369-2 (PMC13412303; doi:10.1186/s40795-026-01369-2)
Supplement: Supplementary file 3 — Additional file 3: Table 2. District-level prevalence of inadequate apparent intakes for vitamins A, C, E, B2, B3, B6, B9, and B12, with 95% confidence intervals. [file 40795_2026_1369_MOESM3_ESM.docx]

**Additional file 3**

**Additional Table 2:** District-level prevalence of inadequate apparent intakes for vitamins A, C, E, B2, B3, B6, B9, and B12, with 95% confidence intervals

| **District** | **Households**  **(n)** | **Vitamin A (RAE)** | **Vitamin C** | **Vitamin E** | **Vitamin B2** | **Vitamin B3** | **Vitamin B6** | **Vitamin B9** | **Vitamin B12** |
| --- | --- | --- | --- | --- | --- | --- | --- | --- | --- |
|  |  | **% (95% CI)** | | | | | | | |
| **Northern region** |  |  |  |  |  |  |  |  |  |
| Chitipa | 384 | 45.8 (36.1–55.6) | 60.1 (50.9–69.4) | 58.9 (52.3–65.4) | 93.0 (89.2–96.8) | 51.6 (45.9–57.3) | 34.1 (29.5–38.6) | 27.2 (22.3–32.0) | 79.8 (74.3–85.3) |
| Karonga | 384 | 48.5 (41.1–55.8) | 72.1 (64.1–80.1) | 71.6 (64.4–78.7) | 92.0 (89.6–94.4) | 64.9 (58.5–71.3) | 44.1 (37.5–50.7) | 38.2 (31.1–45.3) | 63.2 (54.7–71.8) |
| Nkhata-bay | 336 | 29.3 (20.0–38.6) | 32.6 (23.9–41.3) | 54.1 (44.5–63.7) | 66.0 (57.8–74.2) | 33.6 (25.2–41.9) | 18.6 (11.9–25.4) | 11.2 (5.6–16.8) | 48.3 (39.6–56.9) |
| Rumphi | 332 | 32.4 (23.3–41.4) | 47.4 (37.8–56.9) | 45.9 (37.3–54.4) | 83.5 (77.8–89.2) | 41.2 (32.2–50.2) | 21.7 (16.1–27.3) | 23.4 (17.3–29.5) | 62.2 (54.4–69.9) |
| Mzimba | 323 | 39.8 (31.8–47.8) | 56.6 (48.6–64.7) | 65.4 (56.8–73.9) | 91.2 (85.8–96.7) | 63.8 (58.5–69.1) | 45.4 (34.7–56.2) | 42.2 (35.7–48.7) | 72.2 (67.6–76.8) |
| Mzuzu city | 384 | 19.7 (14.4–25.1) | 41.9 (35.9–48.0) | 45.7 (39.2–52.3) | 74.3 (68.4–80.2 | 39.6 (34.2–44.9) | 25.7 (22.2–29.2) | 20.6 (16.9–24.3) | 42.5 (36.0–48.9) |
| **Central region** |  |  |  |  |  |  |  |  |  |
| Kasungu | 384 | 58.0 (48.4–67.6) | 66.5 (61.1–71.9) | 75.4 (69.8–81.0) | 97.1 (95.7–98.4) | 69.4 (64.0–74.7) | 47.4 (40.4–54.3) | 45.5 (37.5–53.5) | 87.4 (83.5–91.3) |
| Nkhotakota | 356 | 51.8 (41.8–61.8) | 64.7 (58.0–71.4) | 66.5 (58.1–75.0) | 87.7 (83.7–91.6) | 60.8 (52.9–68.6) | 28.7 (24.0–33.3) | 36.4 (30.2–42.6) | 77.9 (71.4–84.3) |
| Tchisi | 336 | 60.7 (51.4–70.0) | 56.2 (48.5–63.9) | 71.2 (63.5–79.0) | 93.6 (89.5–97.7) | 61.5 (54.0–69.0) | 44.5 (35.8–53.2) | 46.0 (36.1–55.9) | 88.3 (83.4–93.2) |
| Dowa | 336 | 54.4 (45.3–63.5) | 66.8 (55.8–77.7) | 70.6 (63.5–77.6) | 96.0 (93.6–98.3) | 72.2 (65.9–78.5) | 53.2 (43.3–63.1) | 51.0 (43.0–59.1) | 89.9 (85.8–94.0) |
| Salima | 368 | 52.8 (43.8–61.9) | 71.7 (63.2–80.1) | 66.5 (57.5–75.5) | 90.4 (87.4–93.4) | 64.0 (52.1–76.0) | 47.4 (40.0–54.8) | 44.9 (36.2–53.6) | 84.2 (77.7–90.7) |
| Lilongwe non-city | 574 | 51.1 (43.8–58.4) | 59.7 (55.1–64.3) | 66.4 (60.3–72.5) | 92.7 (89.9–95.5) | 66.7 (59.9–73.4) | 45.5 (40.3–50.7) | 45.8 (39.6–51.9) | 87.4 (82.7–92.0) |
| Lilongwe city | 541 | 18.1 (13.0–23.3) | 55.0 (48.7–61.2) | 50.2 (43.1–57.2) | 84.3 (79.5–89.0) | 47.6 (40.2–55.0) | 28.6 (22.5–34.7) | 19.4 (15.3–23.4) | 57.2 (51.8–62.5) |
| Mchinji | 352 | 58.2 (48.5–67.9) | 59.1 (53.0–65.2) | 76.1 (68.2–84.0) | 94.7 (92.3–97.2) | 67.2 (61.0–73.4) | 51.5 (44.3–58.7) | 45.9 (37.0–54.8) | 91.0 (86.8–95.1) |
| Dedza | 352 | 55.3 (44.9–65.7 | 62.1 (53.4–70.8) | 68.2 (59.7–76.7) | 95.8 (93.4–98.2) | 67.5 (59.3–75.6) | 40.4 (34.1–46.7) | 43.9 (36.9–50.9) | 90.5 (87.0–94.1) |
| Ntcheu | 349 | 53.2 (44.1–62.3) | 52.7 (45.0–60.4) | 66.9 (58.1–75.7) | 91.7 (88.4–95.1) | 63.8 (56.3–71.2) | 40.9 (33.7–48.1) | 39.5 (34.6–44.5) | 94.6 (91.8–97.5) |
| **Southern region** |  |  |  |  |  |  |  |  |  |
| Mangochi | 382 | 41.5 (32.3–50.7) | 47.7 (41.6–53.9) | 55.4 (47.8–63.0) | 92.3 (89.6–95.0) | 57.7 (50.4–65.0) | 31.3 (23.9–38.7) | 33.9 (26.3–41.5) | 75.3 (69.0–81.6) |
| Machinga | 353 | 42.9 (34.9–50.9) | 50.7 (44.6–56.8) | 59.6 (50.4–68.9) | 92.2 (90.1–94.3) | 61.0 (56.1–65.9) | 30.7 (24.5–36.9) | 39.8 (32.0–47.6) | 76.6 (71.4–81.7) |
| Zomba non-city | 352 | 47.9 (38.6–57.2) | 49.6 (42.1–57.2) | 52.8 (44.2–61.4) | 82.6 (77.5–87.7) | 44.2 (36.6–51.8) | 21.7 (16.0–27.4) | 26.8 (20.2–33.5) | 69.1 (64.1–74.1) |
| Zomba city | 332 | 17.9 (12.5–23.2) | 33.8 (28.4–39.2) | 33.3 (26.6–39.9) | 65.5 (60.9–70.1) | 28.7 (23.1–34.3) | 14.4 (10.3–18.6) | 13.6 (8.5–18.6) | 47.0 (41.4–52.7) |
| Chiradzulu | 351 | 43.8 (34.7–52.9 | 48.6 (41.8–55.3) | 52.9 (44.9–60.8) | 80.9 (75.7–86.0) | 41.3 (33.2–49.4) | 17.7 (11.2–24.1) | 22.8 (17.2–28.3) | 66.6 (56.6–76.6) |
| Blantyre non-city | 367 | 43.0 (33.8–52.1) | 52.4 (44.7–60.2) | 54.4 (48.2–60.7) | 85.8 (82.6–89.0) | 52.5 (47.7–57.4) | 29.1 (23.0–35.3) | 27.0 (19.6–34.3) | 70.2 (64.6–75.8) |
| Blantyre city | 352 | 21.7 (14.9–28.5) | 47.4 (41.3–53.4) | 44.5 (38.9–50.0) | 76.1 (71.0–81.1) | 39.7 (33.2–46.2) | 23.3 (17.4–29.2) | 16.1 (12.4–19.8) | 47.1 (41.3–52.9) |
| Mwanza | 319 | 44.6 (34.7–54.5) | 42.0 (32.0–52.0) | 62.8 (55.3–70.3) | 91.7 (88.6–94.7) | 63.6 (54.1–73.0) | 39.5 (32.6–46.3) | 34.7 (21.4–48.1) | 75.2 (66.0–84.5) |
| Thyolo | 384 | 47.5 (38.2–56.8) | 45.7 (37.6–53.7) | 57.9 (51.8–64.1) | 88.2 (84.2–92.2) | 53.1 (47.5–58.8) | 19.3 (14.7–23.9) | 26.0 (19.2–32.7) | 72.2 (66.7–77.7) |
| Mulanje | 368 | 53.7 (45.2–62.1 | 49.3 (41.0–57.5) | 57.6 (50.6–64.6) | 84.1 (79.0–89.1) | 52.8 (45.9–59.8) | 17.7 (12.2–23.3) | 28.1 (21.1–35.2) | 69.0 (61.9–76.0) |
| Phalombe | 352 | 60.6 (53.4–67.8) | 65.8 (60.6–71.0) | 60.3 (55.1–65.5) | 89.6 (87.0–92.1) | 56.5 (50.7–62.3) | 22.5 (17.7–27.4) | 33.5 (25.5–41.4) | 66.7 (61.5–71.8) |
| Chikwawa | 352 | 64.2 (54.4–74.0) | 69.7 (62.8–76.6) | 72.8 (67.1–78.6) | 85.1 (80.0–90.2) | 58.7 (47.8–69.6) | 25.0 (19.5–30.5) | 20.0 (12.6–27.4) | 84.1 (79.0–89.2) |
| Nsanje | 351 | 61.9 (53.2–70.5) | 61.6 (54.2–69.0) | 64.2 (56.8–71.7) | 82.8 (77.9–87.6) | 54.0 (47.4–60.7) | 22.5 (17.2–27.9) | 19.5 (11.1–27.9) | 79.9 (73.9–85.8) |
| Balaka | 367 | 50.8 (42.5–59.1) | 52.7 (45.3–60.0) | 65.2 (58.8–71.7) | 90.5 (86.6–94.4) | 63.9 (56.8–71.1) | 33.3 (26.5–40.2) | 34.4 (26.5–42.3) | 78.4 (71.3–85.4) |
| Neno | 319 | 33.3 (17.6–49.0) | 30.8 (24.0–37.7) | 48.1 (33.1–63.0) | 87.8 (84.0–91.7) | 49.3 (40.9–57.7) | 28.7 (23.4–34.1) | 20.7 (12.7–28.7) | 74.1 (67.9–80.4) |

**Note:** Prevalence and 95% CI are weighted
