# Supplementary material for: The risk of dietary multiple micronutrient inadequacies is widespread and geographically varied in Malawi
Source: BMC Nutr. 2026 May 25;12:147. doi: 10.1186/s40795-026-01369-2 (PMC13412303; doi:10.1186/s40795-026-01369-2)
Supplement: Supplementary file 4 — Additional file 4: Table 3: District-level prevalence of inadequate apparent intake for minerals (Ca, Fe, Se, and Zn), with 95% confidence intervals. [file 40795_2026_1369_MOESM4_ESM.docx]

**Additional file 4**

**Additional Table 3:** District-level prevalence of inadequate apparent intake for minerals (Ca, Fe, Se, and Zn), with 95% confidence intervals

| **District** | **Households (n)** | **Ca** | **Fe** | **Se** | **Zn** |
| --- | --- | --- | --- | --- | --- |
|  |  | **% (95% CI)** | | | |
| **Northern region** |  |  |  |  |  |
| Chitipa | 384 | 89.9 (86.7–93.1) | 77.4 (72.4 – 82.4) | 47.5 (38.8–56.1) | 73.6 (67.2–80.0) |
| Karonga | 384 | 76.9 (69.0–84.8) | 82.6 (79.1 – 86.1) | 43.2 (36.5–50.0) | 73.6 (68.2–79.1) |
| Nkhata-bay | 336 | 44.7 (33.6–55.8) | 65.9 (60.9 – 70.8) | 38.5 (29.5–47.5) | 47.4 (38.0–56.7) |
| Rumphi | 332 | 80.0 (74.3–85.6) | 66.4 (62.2 – 70.6) | 27.4 (21.9–32.9) | 63.1 (55.1–71.1) |
| Mzimba | 323 | 86.2 (79.6–92.8) | 77.2 (71.0 – 83.4) | 54.5 (47.1–62.0) | 76.6 (71.1–82.1) |
| Mzuzu city | 384 | 78.3 (72.9–83.7) | 67.8 (64.0 – 71.7) | 34.6 (29.2–40.0) | 56.8 (52.2–61.4) |
| **Central region** |  |  |  |  |  |
| Kasungu | 384 | 93.9 (89.2–98.7) | 82.5 (78.6 – 86.5) | 64.2 (60.0–68.4) | 75.7 (70.6–80.9) |
| Nkhotakota | 356 | 84.5 (78.5–90.6) | 79.7 (76.1 – 83.3) | 55.8 (49.9–61.8) | 68.2 (62.6–73.8) |
| Tchisi | 336 | 92.3 (87.0–97.6) | 80.5 (74.2 – 86.8) | 55.8 (45.9–65.8) | 74.2 (67.9–80.5) |
| Dowa | 336 | 96.1 (94.1–98.1) | 84.6 (80.8 – 88.4) | 68.6 (62.0–75.3) | 81.5 (77.4–85.7) |
| Salima | 368 | 91.1 (86.3–95.8) | 82.6 (77.9 – 87.4) | 65.9 (57.6–74.2) | 78.1 (70.7–85.4) |
| Lilongwe non-city | 574 | 93.8 (91.3–96.4) | 81.3 (77.9 – 84.7) | 66.6 (62.4–70.8) | 79.6 (76.0–83.1) |
| Lilongwe city | 541 | 89.4 (86.2–92.5) | 79.3 (75.3 – 83.3) | 50.2 (44.2–56.2) | 71.8 (66.4–77.3) |
| Mchinji | 352 | 94.9 (92.7–97.1) | 87.5 (84.7 – 90.2) | 67.4 (62.7–72.1) | 80.4 (75.9–84.9) |
| Dedza | 352 | 97.0 (94.8–99.2) | 83.3 (76.9 – 89.8) | 62.0 (55.1–68.9) | 78.7 (72.9–84.5) |
| Ntcheu | 349 | 96.2 (94.6–97.9) | 78.8 (72.9 – 84.6) | 55.1 (47.5–62.7) | 74.9 (68.7–81.0) |
| **Southern region** |  |  |  |  |  |
| Mangochi | 382 | 81.3 (76.3–86.2) | 73.7 (68.5 – 78.9) | 51.6 (43.3–59.8) | 70.6 (65.1–76.0) |
| Machinga | 353 | 86.7 (81.6–91.7) | 71.4 (65.4 – 77.4) | 49.9 (45.3–54.5) | 65.6 (62.0–69.2) |
| Zomba non-city | 352 | 86.8 (82.6–91.0) | 64.2 (58.4 – 70.0) | 36.1 (28.6–43.7) | 52.6 (45.7–59.6) |
| Zomba city | 332 | 74.8 (69.9–79.7) | 62.9 (59.5 – 66.3) | 27.0 (22.7–31.2) | 43.0 (38.5–47.5) |
| Chiradzulu | 351 | 86.9 (82.4–91.4) | 60.3 (54.6 – 66.0) | 30.5 (23.6–37.3) | 45.5 (38.7–52.2) |
| Blantyre non-city | 367 | 85.4 (81.5–89.2) | 68.3 (65.0 – 71.6) | 41.1 (36.0–46.2) | 63.0 (58.6–67.3) |
| Blantyre city | 352 | 80.9 (76.7–85.1) | 71.1 (67.3 – 75.0) | 35.2 (29.8–40.5) | 63.7 (58.3–69.2) |
| Mwanza | 319 | 88.5 (84.6–92.4) | 75.7 (71.0 – 80.4) | 53.1 (44.7–61.5) | 78.2 (74.4–82.0) |
| Thyolo | 384 | 86.5 (81.1–91.9) | 68.5 (64.6 – 72.5) | 39.5 (33.7–45.4) | 55.6 (49.9–61.3) |
| Mulanje | 368 | 85.8 (80.0–91.6) | 66.9 (62.6 – 71.1) | 44.9 (36.9–52.8) | 53.9 (46.9–60.9) |
| Phalombe | 352 | 91.0 (87.9–94.0) | 68.0 (64.8 – 71.3) | 42.9 (36.9–48.8) | 54.1 (49.3–58.9) |
| Chikwawa | 352 | 89.3 (86.4–92.1) | 52.6 (43.2 – 62.0) | 32.0 (22.8–41.2) | 59.8 (52.1–67.4) |
| Nsanje | 351 | 85.9 (81.6–90.1) | 56.7 (50.5 – 62.9) | 36.6 (28.9–44.3) | 54.6 (47.2–62.1) |
| Balaka | 367 | 88.2 (84.1–92.2) | 74.3 (69.9 – 78.7) | 55.3 (49.2–61.4) | 69.2 (62.6–75.8) |
| Neno | 319 | 86.0 (82.6–89.4) | 68.4 (63.7 – 73.0) | 43.5 (38.3–48.7) | 64.8 (59.6–69.9) |

**Note:** Prevalence and 95% CI are weighted
