# Supplementary material for: The risk of dietary multiple micronutrient inadequacies is widespread and geographically varied in Malawi
Source: BMC Nutr. 2026 May 25;12:147. doi: 10.1186/s40795-026-01369-2 (PMC13412303; doi:10.1186/s40795-026-01369-2)
Supplement: Supplementary file 5 — Additional file 5: Table 4: Median apparent energy intake per day per adult female equivalent disaggregated by residence and district. [file 40795_2026_1369_MOESM5_ESM.docx]

**Additional file 5**

**Additional Table 4:** Median apparent energy intake per day per adult female equivalent disaggregated by residence and district

| **Population** | **Households** | **Apparent energy intake (kcal)** | |
| --- | --- | --- | --- |
|  |  | **Median apparent intake/day per AFE** | **IQR** |
| **Residence** |  |  |  |
| Rural | 9342 | 1876 | 1316 – 2704 |
| Urban | 2090 | 2250 | 1624 – 3053 |
| **Administrative region and their districts** |  |  |  |
| **Northern region (overall)** |  | 2130 | 1588 – 2957 |
| Chitipa | 384 | 2228 | 1615 – 2878 |
| Karonga | 384 | 2171 | 1594 – 2913 |
| Nkhata-bay | 336 | 2742 | 1915 – 3846 |
| Rumphi | 332 | 2537 | 1893 – 3380 |
| Mzimba | 323 | 1825 | 1399 – 2552 |
| Mzuzu city | 384 | 2315 | 1645 – 3438 |
| **Central region (overall)** |  | 1725 | 1205 – 2502 |
| Kasungu | 384 | 1672 | 1131 – 2269 |
| Nkhotakota | 356 | 2045 | 1503 – 2948 |
| Tchisi | 336 | 1921 | 1329 – 2546 |
| Dowa | 336 | 1543 | 1109 – 2386 |
| Salima | 368 | 1538 | 1059 – 2382 |
| Lilongwe non-city | 574 | 1609 | 1163 – 2271 |
| Lilongwe city | 541 | 2117 | 1562 – 2888 |
| Mchinji | 352 | 1634 | 1109 – 2400 |
| Dedza | 352 | 1666 | 1170 – 2430 |
| Ntcheu | 349 | 1769 | 1225 – 2621 |
| **Southern region (overall)** |  | 2080 | 1464 – 2925 |
| Mangochi | 382 | 1859 | 1302 – 2687 |
| Machinga | 353 | 1814 | 1265 – 2527 |
| Zomba non-city | 352 | 2184 | 1678 – 3158 |
| Zomba city | 332 | 2753 | 1995 – 3901 |
| Chiradzulu | 351 | 2452 | 1630 – 3355 |
| Blantyre non-city | 367 | 2138 | 1458 – 2954 |
| Blantyre city | 352 | 2393 | 1764 – 3078 |
| Mwanza | 319 | 1809 | 1304 – 2624 |
| Thyolo | 384 | 2199 | 1596 – 3025 |
| Mulanje | 368 | 2166 | 1549 – 3243 |
| Phalombe | 352 | 2015 | 1389 – 2860 |
| Chikwawa | 352 | 1901 | 1452 – 2628 |
| Nsanje | 351 | 2059 | 1439 – 3061 |
| Balaka | 367 | 1715 | 1209 – 2583 |
| Neno | 319 | 2061 | 1513 – 2653 |

IQR, interquartile range
